# Blockade of Thrombopoietin Reduces Organ Damage in Experimental Endotoxemia and Polymicrobial Sepsis

**DOI:** 10.1371/journal.pone.0151088

**Published:** 2016-03-10

**Authors:** Alessandra Cuccurullo, Elisabetta Greco, Enrico Lupia, Paolo De Giuli, Ornella Bosco, Erica Martin-Conte, Tiziana Spatola, Emilia Turco, Giuseppe Montrucchio

**Affiliations:** 1 Department of Medical Sciences, University of Turin, Turin, Italy; 2 Anatomia Patologica, San Lazzaro Hospital, Alba, Italy; 3 Department of Anaesthesiology and Critical Care, University of Turin, Turin, Italy; 4 Department of Genetics, Biology and Biochemistry, University of Turin, Turin, Italy; University of Torino, ITALY

## Abstract

**Background and Purpose:**

Thrombopoietin (TPO), a growth factor primarily involved in thrombopoiesis may also have a role in the pathophysiology of sepsis. In patients with sepsis, indeed, TPO levels are markedly increased, with disease severity being the major independent determinant of TPO concentrations. Moreover, TPO increases and correlates with ex vivo indices of platelet activation in patients with burn injury upon sepsis development, and may contribute to depress cardiac contractility in septic shock. Still, the role of TPO in sepsis pathophysiology remains controversial, given the protective role of TPO in other experimental disease models, for instance in doxorubicin-induced cardiotoxicity and myocardial ischemia/reperfusion injury. The aim of our study was to define the contribution of TPO in the development of organ damage induced by endotoxemia or sepsis, and to investigate the effects of inhibiting TPO in these conditions.

**Methods:**

We synthesized a chimeric protein able to inhibit TPO, mTPOR-MBP, and studied its effect in two murine experimental models, acute endotoxemia and cecal ligation and puncture (CLP) model.

**Results:**

In both models, TPO levels markedly increased, from 289.80±27.87 pg/mL to 465.60±45.92 pg/mL at 3 hours in the LPS model (*P*<0.01), and from 265.00±26.02 pg/mL to 373.70±26.20 pg/mL in the CLP model (*P*<0.05), respectively. Paralleling TPO levels, also platelet-monocyte aggregates increased, from 32.86±2.48% to 46.13±1.39% at 3 hours in the LPS model (*P*<0.01), and from 43.68±1.69% to 56.52±4.66% in the CLP model (*P*<0.05). Blockade of TPO by mTPOR-MBP administration reduced histological damage in target organs, namely lung, liver, and gut. In particular, neutrophil infiltration and lung septal thickening were reduced from a score of 1.86±0.34 to 0.60±0.27 (*P*<0.01) and from 1.43±0.37 to 0.40±0.16 (*P*<0.05), respectively, in the LPS model at 3 hours, and from a score of 1.75±0.37 to 0.38±0.18 (*P*<0.01) and from 1.25±0.31 to 0.13±0.13 (*P*<0.001), respectively, in the CLP model. Similarly, the number of hepatic microabscesses was decreased from 14.14±1.41 to 3.64±0.56 in the LPS model at 3 hours (*P*<0.001), and from 1.71±0.29 to 0.13±0.13 in the CLP model (*P*<0.001). Finally, the diameter of intestinal villi decreased from 90.69±3.95 μm to 70.74±3.60 μm in the LPS model at 3 hours (*P*<0.01), and from 74.29±4.29 μm to 57.50±1.89 μm in the CLP model (*P*<0.01). This protective effect was associated with the blunting of the increase in platelet-monocyte adhesion, and, on the contrary, with increased platelet-neutrophil aggregates in the circulation, which may be related to decreased neutrophil sequestration into the inflamed tissues. Conversely, circulating cytokine levels were not significantly changed, in both models, by mTPOR-MBP administration.

**Conclusion:**

Our results demonstrate that TPO participates in the development of organ damage induced by experimental endotoxemia or polymicrobial sepsis via a mechanism involving increased platelet-leukocyte adhesion, but not cytokine release, and suggest that blocking TPO may be useful in preventing organ damage in patients affected by systemic inflammatory response or sepsis.

## Introduction

Thrombopoietin (TPO), a growth factor primarily involved in regulating thrombopoiesis [1, 2], has recently emerged as a humoral mediator potentially implicated in the pathogenesis of sepsis [3].

TPO is constitutively produced by liver and kidneys, and cleared from the circulation by platelets upon binding to the TPO receptor c-Mpl, followed by internalization of the bound ligand [1, 2]. Its best characterized action is to stimulate platelet production by acting on megakaryocytic progenitor cells, as well as self-renewal and expansion of hematopoietic stem cells [4]. TPO also acts on cell types other than hematopoietic progenitors, exerting a variety of effects. In particular, TPO, although unable per se to induce platelet aggregation, enhances the response of mature platelets to several agonists [5, 6], and increases platelet-leukocyte adhesion via P-selectin [7], a phenomenon that has been associated with the development of acute coronary syndromes [8]. Moreover, TPO increases oxygen radical release and induces IL-8 production by neutrophils and monocytes [9]. Finally, TPO elicits various effects in other c-Mpl-expressing cells, including endothelial [10, 11], cardiac [12], and neuronal cells [13, 14].

Several studies have previously reported that TPO levels are increased after endotoxin infusion and in patients with sepsis [3], especially those with severe sepsis and septic shock [15]. TPO also increases in patients with burn injury upon sepsis development, and correlates with ex vivo indices of platelet activation, namely platelet P-selectin expression and increased platelet-monocyte adhesion [16], suggesting that TPO, by favoring platelet activation, may contribute to trigger multi-organ damage in these diseases [3]. Finally, TPO may depress cardiac contractility in septic shock, since it inhibits adrenergic receptor signal transduction in myocardiocytes, and cooperates with TNF-α and IL-1β to the cardio-depressant activity exerted in vitro by serum of septic shock patients [12]. Similar results, indicating TPO as a mediator of organ damage, have also been reported in experimental models of acute lung injury [17] and acute pancreatitis [18]. However, other studies rather support a protective effect of TPO, for instance against cardiotoxicity induced by doxorubicin [19] and myocardial ischemia/reperfusion injury [20].

In this study, we aimed to define the role of TPO in the development of organ damage induced by experimental endotoxemia or polymicrobial sepsis, and to investigate the effects of inhibiting TPO in these conditions. To this end, we generated and characterized for its inhibiting activity against TPO a soluble recombinant chimeric protein, mTPOR-MBP, composed of the extracellular portion of mouse TPO receptor mTPOR/cMpl fused to the maltose-binding protein (MBP) in order to increase its solubility. We then studied whether TPO inhibition by mTPOR-MBP may reduce organ damage in two widely used experimental models, one mimicking a systemic inflammatory response syndrome, induced by i.p. injection of lipopolysaccharides (LPS), the other a model of abdominal infection leading to polymicrobial sepsis, represented by the cecal ligation and puncture (CLP) model.

## Materials and Methods

### Synthesis of mTPOR-MBP

We generated mTPOR-MBP by fusing the extracellular portion of mouse TPO receptor mTPOR/c-Mpl to maltose-binding protein (MBP). The DNA fragment encoding murine c-Mpl-extracellular domain (26–491) was amplified by PCR through high-fidelity DNA polymerase (Stratagene, La Jolla, CA), using 300 ng of murine c-Mpl cDNA (K.K. Dnaform, Ibarak–Japan) as a template. The following primers were used: Forward 5’CCGGAATTCATGAGCCAAGATGTCTTCTTGCTGGCCT3’; Reverse 5’TGCTCTAGATTACAGGAGCAGAGCAGTCAC3’, carrying, at the 5’-end, the sequence for EcoRI and XbaI restriction enzymes, respectively. The amplification product was first digested with EcoRI and XbaI and purified on agarose gel. The 1425 bp PCR product was then sub-cloned into the expression vector pMal-c2 digested with EcoRI and XbaI (New England Biolabs, Ipswich, MA), downstream and in frame with the *malE* gene encoding MBP. Escherichia coli strain BL21(DE3)pLysS cells (Biocompare, Inc., South San Francisco, CA) was transformed with the vector. Protein expression was induced with 0.1 mM isopropyl β-D-1-thiogalactopyranoside (IPTG) for 3 h at 37°C. Thereafter, cells were collected by centrifugation, frozen at -20°C, then thawed at 37°C, and lysed by sonication. The soluble fraction containing mTPOR-MBP fusion protein was loaded on a pre-packed amylose affinity column (New England Biolabs), and elution was performed with the same buffer supplemented with 10 mM maltose. mTPOR-MBP was finally dialysed against phosphate-buffered saline (PBS) and stored at -70°C.

Protein was analysed by SDS-PAGE on 8% polyacrylamide gel under reducing conditions, and transferred onto nitrocellulose. The membrane was blocked with 5% BSA in Tris-buffered solution containing Tween-20 (TBS-T) overnight at 4°C, followed by incubation with either anti-murine c-Mpl monoclonal antibody (R&D Systems Inc., Minneapolis, MN), or anti-MBP rabbit polyclonal serum. Blots were probed with peroxidase-conjugated sheep anti-mouse (Amersham, Buckinghamshire, UK), or goat anti-rabbit (Pierce, Rockford, IL) antibodies, as appropriate, and developed with chemiluminescence reagents (PerkinElmer LAS, Boston, MA).

### Characterization of mTPOR-MBP

Binding specificity of mTPOR-MBP with TPO was analysed by dot-blot as previously described [21]. Briefly, recombinant (r) TPO (R&D Systems Inc.) was diluted in PBS buffer (pH 7.4) at a concentration ranging from 3.12 μg/ml to 25 μg/ml. TNF-α and IL-1β (25 μg/ml, Sigma-Aldrich, St Louis, MO) were used as controls. 5 μl of each sample were spotted onto a nitrocellulose membrane, then blocked with 5% BSA in TBS-T buffer for 1 hour. The membrane was incubated with the mTPOR-MBP (0.5 μg/ml) overnight at 4°C, and subsequently with anti-murine c-Mpl/TPOR monoclonal antibody for 2 hours at room temperature. Blots were probed with peroxidase-conjugated sheep anti-mouse antibody and developed with chemiluminescence reagents.

The ability of mTPOR-MBP to block TPO biological activity *in vitro* was tested by using a short-term proliferative assay on the megakaryoblast cell line M-07 [22] and platelet aggregation experiments in platelet-rich plasma (PRP) [5].

For the proliferative assay, 2x10^5^ M-07 cells were plated in a 96-wells microplate and stimulated with rTPO (50–1000 pg/ml), in the presence or absence of mTPOR-MBP (1–20 μg/ml) for 48 hours. Afterwards each well was pulsed with 1 μC of tritiated thymidine (Amersham) allowing incorporation for 16 hours. Cells were then harvested, and thymidine incorporation was determined in a scintillation counter after addition to each vial of 1mL of scintillation liquid.

For platelet aggregation experiments, blood was collected by clean venipuncture using a 19-gauge butterfly infusion set, without venous stasis, from healthy adult donors, who had not taken any medications for at least 2 weeks. Nine volumes of blood were withdrawn in 1 vol of 3.8% trisodium citrate. PRP was prepared by centrifugation for 15 minutes at 180g. Platelet poor plasma (PPP) was obtained by centrifugation at 2,000g for 10 minutes. Platelet aggregation in PRP was evaluated according to the Born's method [23], at 37°C with constant rate of stirring at 1,000 rpm in a lumi-aggregometer (Chronolog, Havertown, PA) using PPP as reference, setting to 0% the light transmission using PRP and to 100% using PPP. The maximal aggregation was quantified according to the Weiss formula [24]. PRP samples were incubated with 100 pg/ml rTPO for 5 minutes, before epinephrine (EPI; Helena Laboratories, Beaumont, TX), as secondary agonist, was added. For each experiment, the concentration of EPI that induced the minimum measurable aggregation was determined (0.1–1 μmol/L). The priming index (PI) was calculated as the response to rTPO and EPI together, divided by the sum of individual responses elicited by rTPO and EPI separately. Using this calculation, a PI > 1 indicated synergism, a PI = 1 indicated additive response, and PI < 1 indicated inhibition [5,16]. In separate experiments, rTPO was pre-incubated with the mTPOR-MBP, or purified MBP as control (2 μg/ml each), for 5 minutes at 37°C; the mixture of rTPO and mTPOR-MBP or purified MBP was added to PRP, further incubated for 5 minutes at 37°C and stimulated with EPI.

In preliminary experiments, mice were injected i.p. with 40 μg of mTPOR-MBP, and, for the next 24 hours, the presence of mTPOR-MBP in plasma was checked. Briefly, blood was drawn by the tail vein, centrifuged at 600g for 10 minutes in order to obtain plasma, and the presence of mTPOR-MBP was detected by western blot analysis, using the anti-MBP rabbit polyclonal serum.

### Experimental models

Three month-old male C57BL/6 mice were used for all experiments (the exact n/group distributions are specified in the figure legends). Mice were kept together in groups of five per cage, housed on a 12 h light-dark diurnal cycle with controlled temperature (21°C to 23°C) and provided with standard rodent diet and water ad libitum.

Acute endotoxemia was induced by i.p. injection with 40 mg/kg of LPS from E. coli 0111:B4 (Sigma-Aldrich) in a volume of 250 μl sterile saline solution [25]. A separate group of animals received an i.p. injection of 40 μg of mTPOR-MBP in a volume of 200 μl sterile saline solution, 10 minutes after LPS administration. As controls, separate groups of mice received the same concentration of mTPOR-MBP alone, MBP alone, and/or sterile saline solution.

To confirm the results obtained with LPS injection, we used a second model of polymicrobial sepsis induced by cecal ligation and puncture (CLP) [26]. Mice were anesthetized with tiletamine (80 mg/kg), and a midline abdominal incision was performed. The cecum was mobilized, ligated below the ileo-cecal valve, and punctured twice with a 25-gauge needle. The abdomen was then closed in two layers, and the mice injected s.c. with 1.0 ml 0.9% saline. A separate group of animals received an i.p. injection of 40 μg of mTPOR-MBP in a volume of 200 μl sterile saline solution, 30 minutes after surgery was completed. As controls, separate groups of mice received the same concentration of MBP alone and/or sterile saline solution. Sham-operated mice were handled in the same manner, except the cecum was neither ligated nor punctured.

This study was carried out in strict accordance with the recommendations in the Guide for the Care and Use of Laboratory Animals of the National Institutes of Health, as well as institutional guidelines and Italian government regulations. The protocol was approved by the Institutional Review Committee for Animal Care and Use at the University of Turin (Protocol Number: 0008619-P). All surgery was performed under anesthesia, and all efforts were made to minimize suffering. In particular, buprenorphine (0.05 mg/kg i.p.) was administered before surgery as well as 6 hours after surgery to reduce postoperative pain, and animals were inspected for signs of illness and/or unusual behavior by research staff at least once per day. Whenever an animal's condition deteriorated (defined by, among other parameters, decreased activity, and rapid weight gain) followed by the appearance of signs of imminent death (i.e. inability to maintain upright position/ataxia/tremor and/or agonal breathing) mice were killed by using deep inhalation anesthesia (isoflurane) followed by an overdose of barbiturate (thiopental). The percentage of premature deaths in both models did not exceed 10%.

### Sample collection and cell counts

At the indicated times (basal, 3, and 6 hours after LPS injection, and after 18 hours in the CLP model), mice were anesthetized with tiletamine (80 mg/kg), and blood samples were collected from the inferior cava vein into tubes containing trisodium citrate (0.11 mol/liter). The examined time-points have been chosen on the basis of preliminary experiments performed in our laboratory, and of similar studies already published in the literature [25, 27, 28]. Blood was used for platelet and white blood cell counting, using an automated counter, for flow cytometry analysis, and to obtain plasma, after centrifugation at 600g for 10 minutes. Soon after, liver and small intestine were rapidly dissected and fixed in 4% neutral buffered formalin for histological analysis. A polyvinyl catheter was then secured in the trachea, and used to instil 4% neutral buffered formalin into the lungs at a hydrostatic pressure of 30 cmH_2_O. Portions of the formalin-distended lungs were harvested and used for histological analysis.

### Histological analysis

Histological damage was evaluated on H&E-stained 5 μm paraffin sections by an expert pathologist (P.D.G.) blind to the sample identity. To measure lung injury, neutrophil infiltration and alveolar-capillary membrane thickening were scored by assigning a subjective value from zero (absent) to three (extensive) scale. In each tissue sample, five random fields were scored, and the mean value was calculated.

To evaluate liver injury, the number of leukocyte microabscesses, i.e. discrete neutrophil infiltrates around the portal veins that derive from neutrophil infiltration within the liver parenchyma [25], was counted in 10 randomly-selected fields.

Gut injury was evaluated by measuring intestinal villus thickness in 5 randomly-selected fields.

### TPO and cytokine measurement

TPO levels in plasma were determined by ELISA (R&D Systems Inc.) according to the manufacturer’s recommendations, with the exception of a 3-fold plasma dilution.

Cytokine (TNF-α, IL-6, and IL-10) concentrations were measured in plasma samples using a fluorescent bead–based multiplex immunoassay (Bio-plex®/Luminex®, Luminex Co., Austin, TX), as previously described [29, 30].

### Flow cytometry

Leukocyte-platelet aggregates ex vivo were analyzed by two-colour staining of whole blood samples. Briefly, blood was collected, diluted 1:1 with Tyrodes’s Hepes-buffered saline (pH 7.4), and incubated 15 minutes at room temperature with a mixture of PE-Cy5-conjugated anti-mouse-CD45 monoclonal antibody (eBioscience, San Diego, CA), which binds all leukocytes, and FITC-conjugated anti-mouse-CD41 (Serotec, Raleigh, U.S.A.) monoclonal antibody, which identifies platelets. Cells were then fixed with 1% paraformaldehyde and erythrocytes were removed by hypotonic lysis. Samples were washed twice with PBS-BSA 0.1% and resuspended in 0.5 ml PBS. Samples were analyzed on a EPICS–XL flow cytometer (Beckman Coulter Corp, Hialeah, FL). Total leukocytes were identified by their positive staining with anti-CD45 in the CD45 versus side scatter dot plot, whereas lymphocyte, neutrophil and monocyte populations were discriminated by size and granularity in the forward scatter versus side scatter dot plot using a logic gate. The percentage of monocyte-platelet or neutrophil-platelet aggregates, therefore co-expressing both markers CD45 and CD41 over the total number of cells counted within the logic gates previously selected was calculated and used as an index of platelet-monocyte or platelet-neutrophil adhesion.

### Analysis of data

Data are expressed as mean ± S.E.M. Comparisons between groups were analyzed by ANOVA followed by Newman-Keul’s multicomparison test or by Student’s *t* test, where appropriate. Relationship between variables was investigated by Spearman rank correlation coefficient.

A *P* value < 0.05 was considered significant.

All statistics were done with GraphPad Prism 4.00 for Windows (GraphPad Software, La Jolla, CA, USA).

## Results

### Synthesis and characterization of mTPOR-MBP

**Fig 1:**
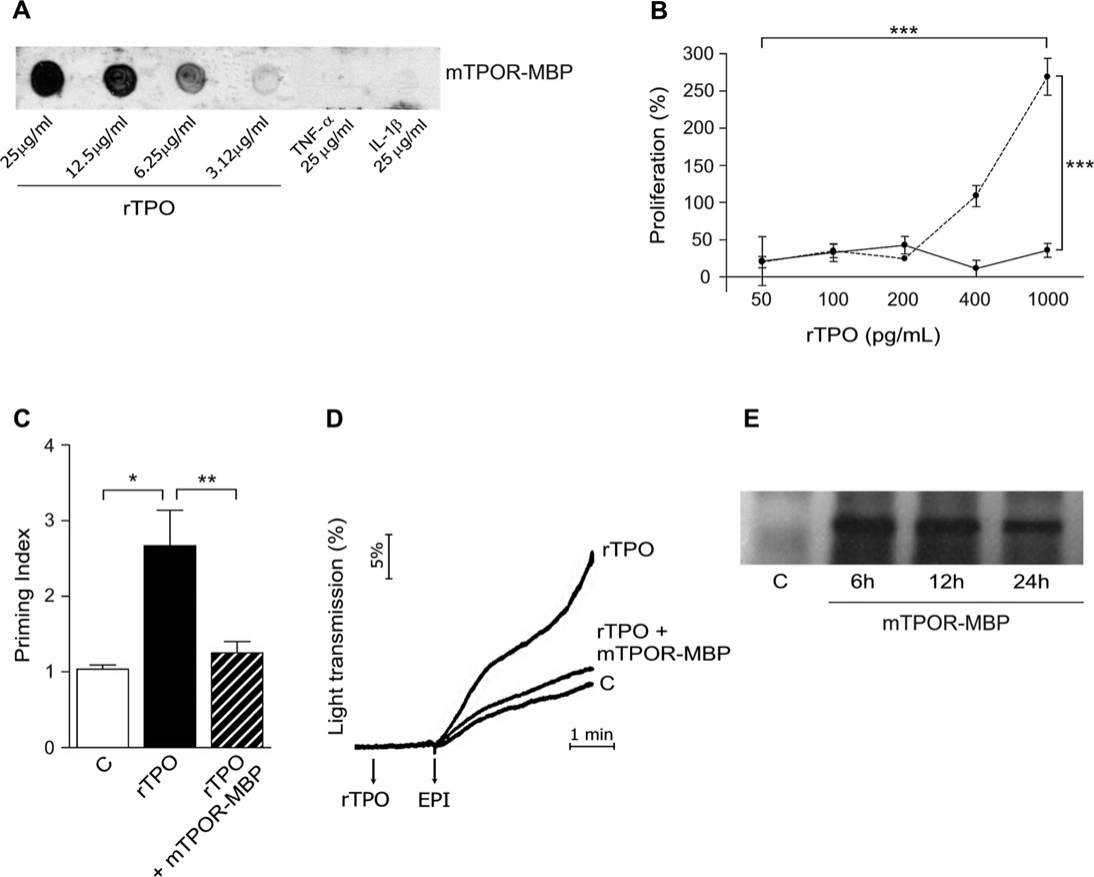
Characterization of the chimeric protein mTPOR-MBP. (A) Dot-Blot analysis of mTPOR-MBP binding specificity. Recombinant(r) TPO, TNF-α and IL-1β were spotted onto a nitrocellulose membrane, then incubated with mTPOR-MBP, and revealed with anti-c-Mpl/TPOR antibody, as described in the Materials and Methods section. Blots are representative of three independent experiments with similar results. (B) Short-term proliferative assay of M-07 cells stimulated with 50–1000 pg/ml rTPO in the presence (continuous line) or absence (dotted line) of 2 μg/ml mTPOR-MBP. Data represent means ± S.E.M. of at least 3 separate experiments with similar results. (C-D) Quantification (C) and representative aggregation traces (D) of the priming effect of 0.5 ng/ml rTPO in the presence or absence of 2 μg/ml mTPOR-MBP on epinephrine (EPI)-induced platelet aggregation in platelet-rich plasma (see Materials and Methods for experimental details). C indicates pre-incubation with vehicle alone (phosphate-buffered saline-PBS). The priming index (PI) reported in panel C was calculated as the response to rTPO and EPI together, divided by the sum of individual responses elicited by rTPO and EPI separately, as detailed in the Materials and Methods section. Light transmission changes (panel D) are recorded as an expression of platelet aggregation after agonist addition, and expressed as percent of maximal value (set to 100% using PPP as reference). Data represent means ± S.E.M. of at least 3 separate experiments with similar results. * *P* < 0.05; ** *P* < 0.01; *** *P* < 0.001. (E) Presence of mTPOR-MBP in the circulation up to 24 hours after i.p injection, as detected by western blot analysis (see Materials and Methods for details).

Aiming to block TPO actions in vivo, we designed and synthesized a chimeric protein, mTPOR-MBP, originated by the fusion of the extracellular portion of mTPOR/c-Mpl and of MBP, used to enhance its solubility. The soluble form of mTPOR-MBP was purified by affinity chromatography using an amylose column, and recognized by antibodies directed against MBP and TPOR/c-Mpl by western blot. mTPOR-MBP showed dose-dependent high-affinity binding for rTPO in dot-blot assay, whereas it did not recognized TNF-α and IL-1β (Fig 1A). In addition, it reduced the proliferation induced by rTPO in M-07 cells (Fig 1B) [22], and the priming effect exerted by rTPO on epinephrine-induced platelet aggregation in PRP (Fig 1, panels C and D) [5, 8]. mTPOR-MBP inhibitory effect on platelet aggregation was dose-dependent, and reached a plateau at a concentration of 1.5–2 μg/ml in the presence of 100 pg/ml of rTPO. mTPOR-MBP alone did not induce any effect neither on M-07 cell proliferation nor on platelet aggregation (not shown). Finally, once-daily administration was chosen on the basis of the presence of mTPOR-MBP in mouse plasma, as detected by Western blot analysis, up to 24 hours after i.p. administration (Fig 1E).

### TPO concentrations increased in experimental endotoxemia and polymicrobial sepsis

**Fig 2:**
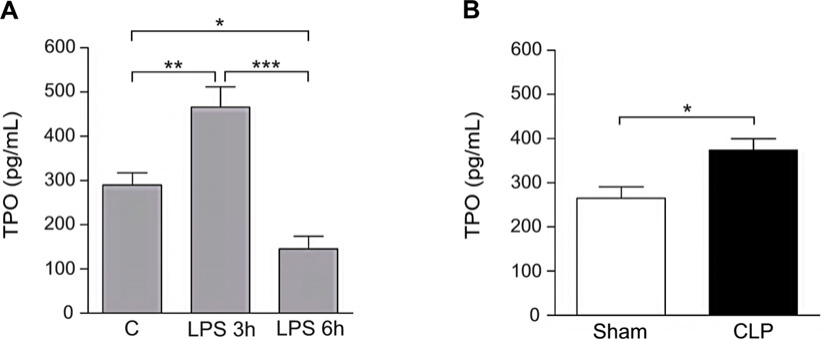
**Plasma thrombopoietin (TPO) levels in the LPS model (Panel A) and in the cecal ligation and puncture (CLP) model (Panel B).** TPO concentrations were measured by ELISA at the indicated time-points in the LPS model and 18 hours after sepsis induction in the CLP model. The number of animals studied per experimental group was as follows: Panel A: Control (C) = 13; LPS 3h = 11; LPS 6h = 6. Panel B: Sham-operated mice = 5; CLP = 6. * *P* < 0.05; ** *P* < 0.01; *** *P* < 0.001.

Previous studies have shown that TPO levels are increased after endotoxin infusion and in patients with sepsis [3, 15]. Here, we analyzed whether TPO levels were also increased in an acute endotoxemia model and in the CLP model. In acute endotoxemia induced by LPS i.p. injection, TPO concentrations increased at 3 hours from LPS administration, both in comparison to basal levels, and to those measured in mice injected with saline, and decreased at 6 hours to concentrations lower than those measured at time 0 and after 3 hours (Fig 2A). Also in the CLP model, TPO levels were significantly increased in septic mice compared to those measured in sham-operated mice (Fig 2B).

### mTPOR-MBP reduces organ damage in experimental endotoxemia and polymicrobial sepsis

It is known that sepsis mortality is strictly related to the development of injury and failure in target organs [31]. In both models investigated, the induction of systemic inflammation or sepsis was associated with the development of severe histological lesions in the target organs, which were significantly reduced by mTPOR-MBP administration.

**Fig 3:**
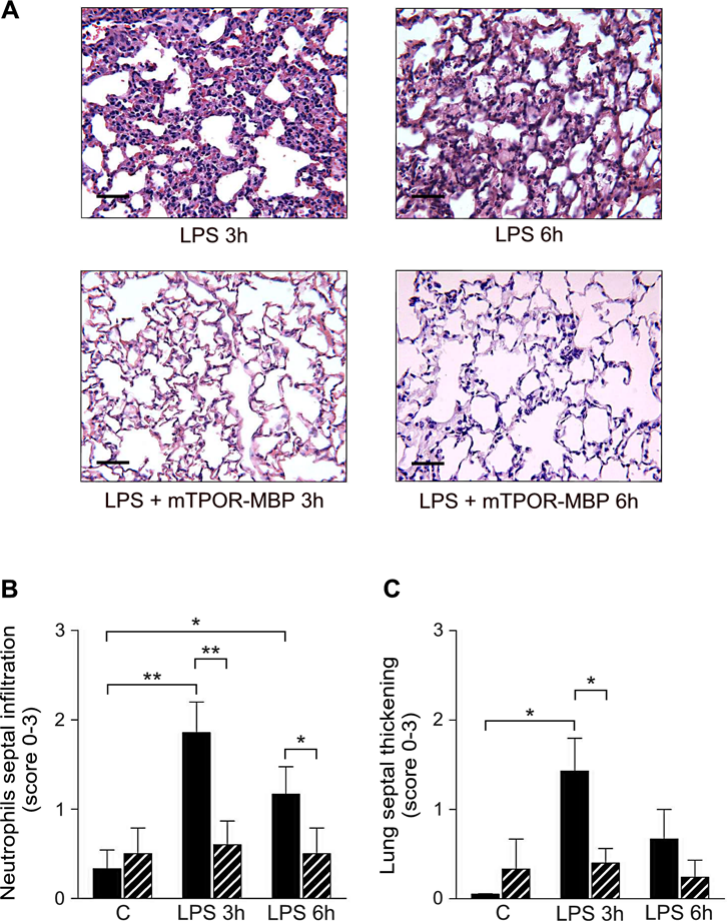
Effect of mTPOR-MBP on the development of lung damage in the LPS model. (A) Representative micrographs of lung histology in LPS-injected mice untreated (upper panel) or treated (lower panel) with mTPOR-MBP at the indicated time-points (see Materials and Methods for experimental details). Original magnification, X200. Black bars, 50 μm. (B) Quantification of neutrophil infiltration within lung septa after LPS administration, in mice treated (dashed columns) or not (black columns) with mTPOR-MBP (see Materials and Methods for methodological details). (C) Quantification of lung septal thickening after LPS administration, in mice treated (dashed columns) or not (black columns) with mTPOR-MBP (see Materials and Methods for methodological details). The number of animals studied per experimental group was as follows: Control (C) = 6; mTPOR-MBP alone = 5; LPS 3h = 7; LPS 3h + mTPOR-MBP = 10; LPS 6h = 6; LPS 6h + mTPOR-MBP = 5. * *P* < 0.05; ** *P* < 0.01.

**Fig 4:**
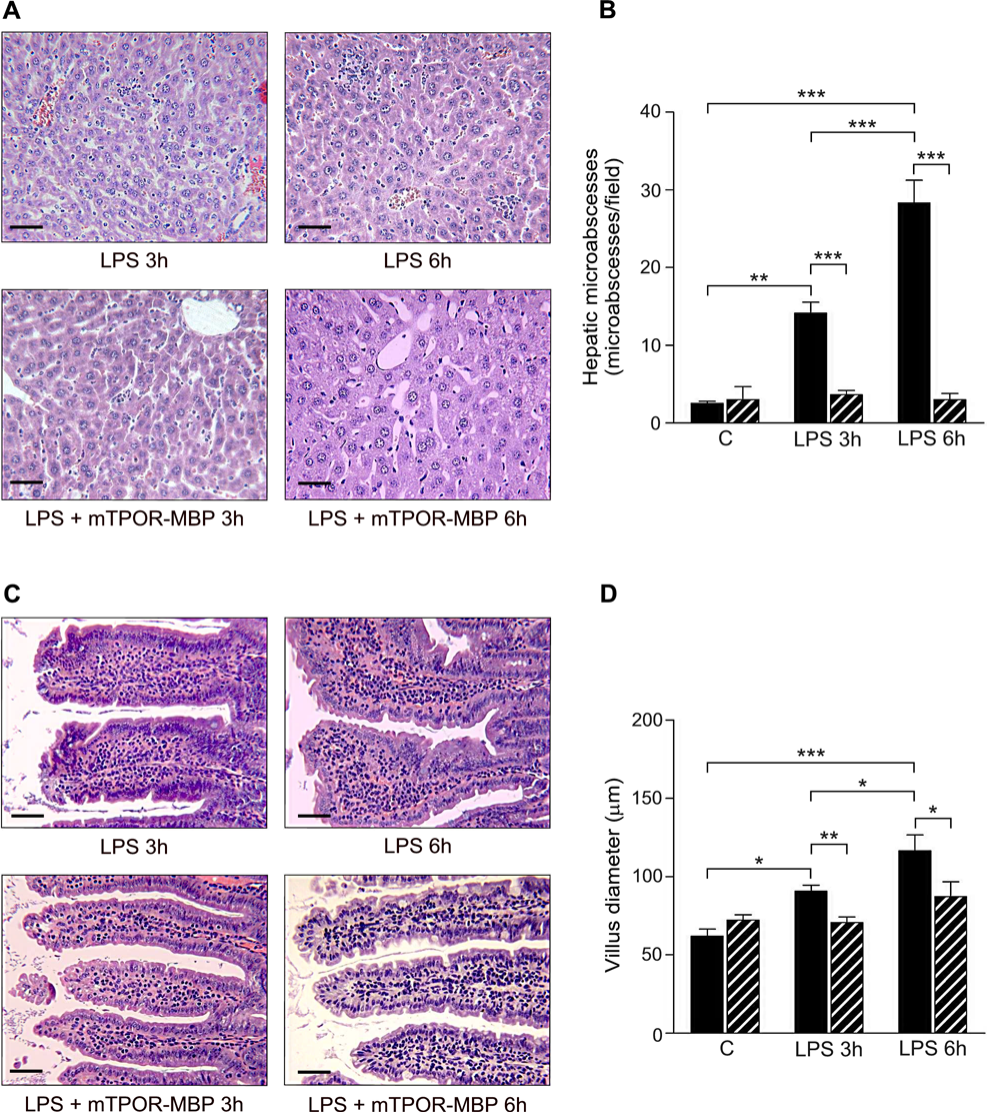
Effect of mTPOR-MBP on the development of liver and intestinal damage in the LPS model. (A) Representative micrographs of liver histology in LPS-injected mice untreated (upper panel) or treated (lower panel) with mTPOR-MBP at the indicated time-points (see Materials and Methods for experimental details). Original magnification, X200. Black bars, 50 μm. (B) Quantification of hepatic micro-abscesses after LPS administration, in mice treated (dashed columns) or not (black columns) with mTPOR-MBP (see Materials and Methods for methodological details). (C) Representative micrographs of gut histology in LPS-injected mice untreated (upper panel) or treated (lower panel) with mTPOR-MBP at the indicated time-points (see Materials and Methods for experimental details). Original magnification, X200. Black bars, 50 μm. (D) Quantification of intestinal villus diameter after LPS administration, in mice treated (dashed columns) or not (black columns) with mTPOR-MBP(see Materials and Methods for methodological details). The number of animals studied per experimental group was as follows: Control (C) = 6; mTPOR-MBP alone = 5; LPS 3h = 7; LPS 3h + mTPOR-MBP = 10; LPS 6h = 6; LPS 6h + mTPOR-MBP = 5. * *P* < 0.05; ** *P* < 0.01; *** *P* < 0.001.

The effect of mTPOR-MBP on the development of organ damage induced by LPS administration was evaluated at 3 and 6 hours (Figs 3 and 4). Lung injury, evaluated by measuring neutrophil infiltration and alveolar septal thickening, was significantly reduced by mTPOR-MBP at both time-points (Fig 3).

Moreover, mTPOR-MBP reduced the number of hepatic micro-abscesses around the centrolobular veins, consequent to massive infiltration of inflammatory cells into the hepatic parenchyma, at both 3 and 6 hours from LPS injection (Fig 4, panels A and B). Finally, mTPOR-MBP prevented the development of intestinal injury, which led to increased diameter of villi, secondary to leukocyte recruitment and edema, at both time-points (Fig 4, panel C and D).

**Fig 5:**
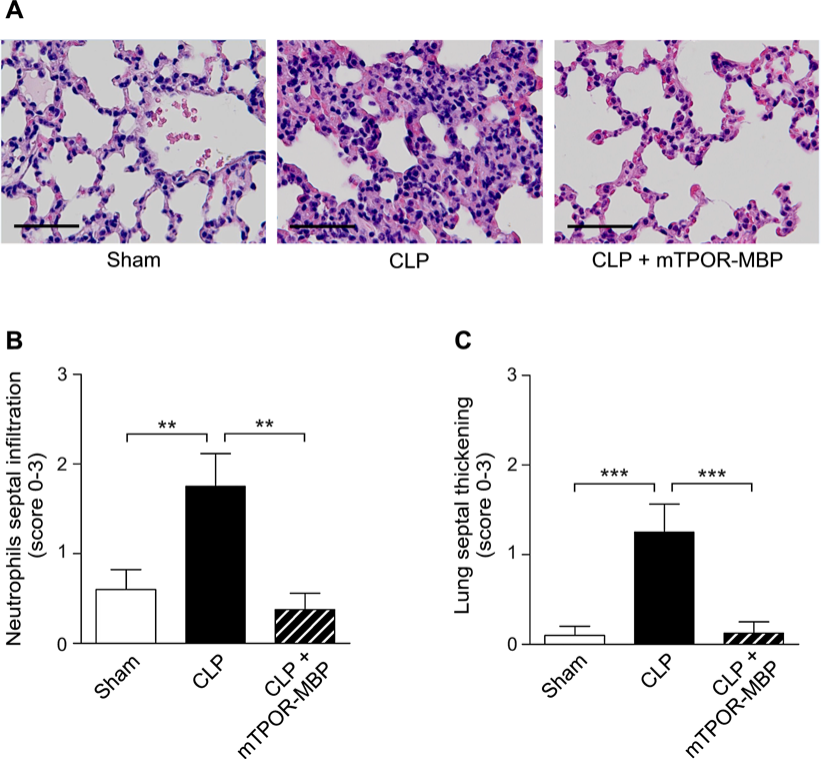
Effect of mTPOR-MBP on the development of lung damage in the cecal ligation and puncture (CLP) model. (A) Representative micrographs of lung histology in sham-operated mice and septic mice 18 hours after CLP, treated or not with mTPOR-MBP (see Materials and Methods for experimental details). Original magnification, X400. Black bars, 50 μm. (B) Quantification of neutrophil infiltration within lung septa in sham-operated mice (white columns) and septic mice 18 hours after CLP, treated (dashed columns) or not (black columns) with mTPOR-MBP (see Materials and Methods for methodological details). (C) Quantification of lung septal thickening in sham-operated mice (white columns) and septic mice 18 hours after CLP, treated (dashed columns) or not (black columns) with mTPOR-MBP (see Materials and Methods for methodological details). The number of animals studied per experimental group was as follows: Sham-operated mice = 10; CLP = 8; CLP + mTPOR-MBP = 8. ** *P* < 0.01; *** *P* < 0.001.

**Fig 6:**
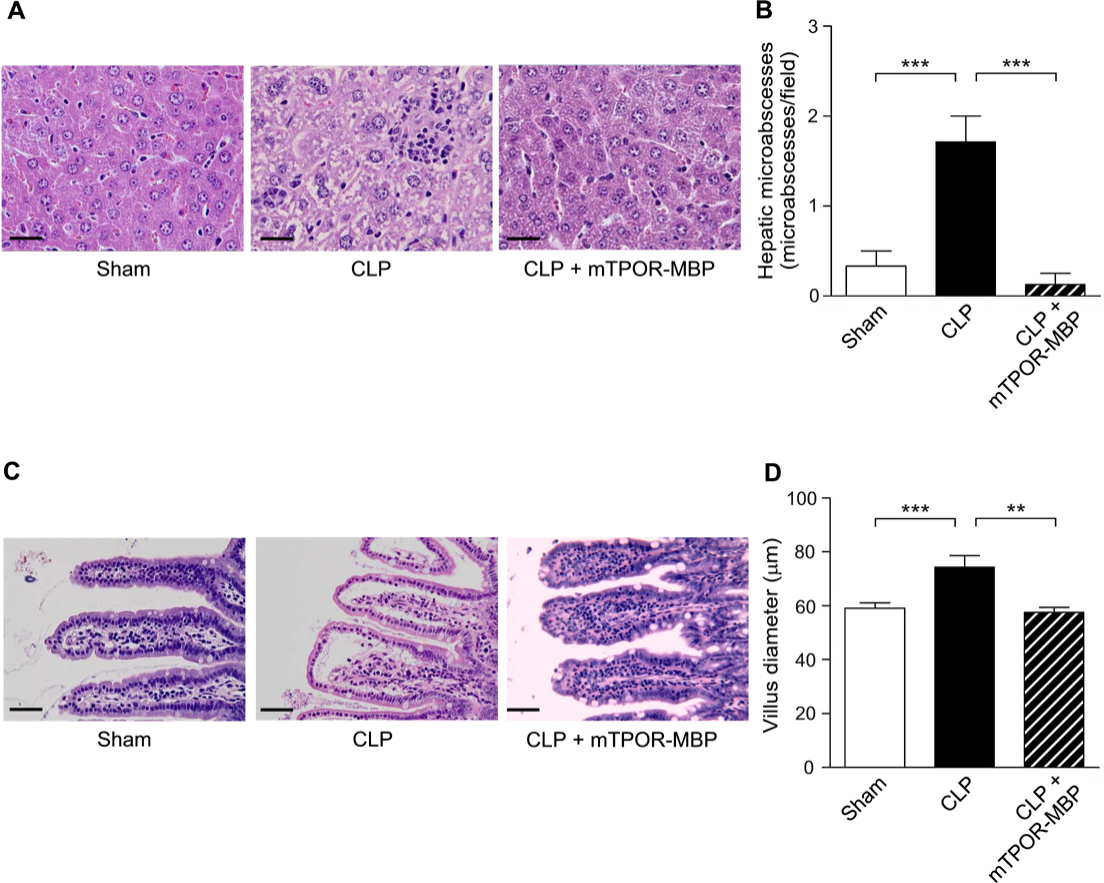
Effect of mTPOR-MBP on the development of liver and intestinal damage in the cecal ligation and puncture (CLP) model. (A) Representative micrographs of liver histology in sham-operated mice and septic mice 18 hours after CLP, treated or not with mTPOR-MBP (see Materials and Methods for experimental details). Original magnification, X200. Black bars, 50 μm. (B) Quantification of hepatic micro-abscesses in sham-operated mice (white columns) and septic mice 18 hours after CLP, treated (dashed columns) or not (black columns) with mTPOR-MBP (see Materials and Methods for methodological details). (C) Representative micrographs of gut histology in sham-operated mice and septic mice 18 hours after CLP, treated or not with mTPOR-MBP (see Materials and Methods for experimental details). Original magnification, X200. Black bars, 50 μm. (D) Quantification of intestinal villus diameter in sham-operated mice (white columns) and septic mice 18 hours after CLP, treated (dashed columns) or not (black columns) with mTPOR-MBP (see Materials and Methods for methodological details). The number of animals studied per experimental group was as follows: Sham-operated mice = 10; CLP = 8; CLP + mTPOR-MBP = 8. * *P* < 0.05; ** *P* < 0.01; *** *P* < 0.001.

Also in the CLP model, mTPOR-MBP significantly reduced the severity of tissue injury induced in lung, liver and gut (Figs 5 and 6).

mTPOR-MBP alone did not show any effect on lung, liver and gut histology in both models (not shown).

### mTPOR-MBP does not affect cytokine release in experimental endotoxemia and polymicrobial sepsis

**Fig 7:**
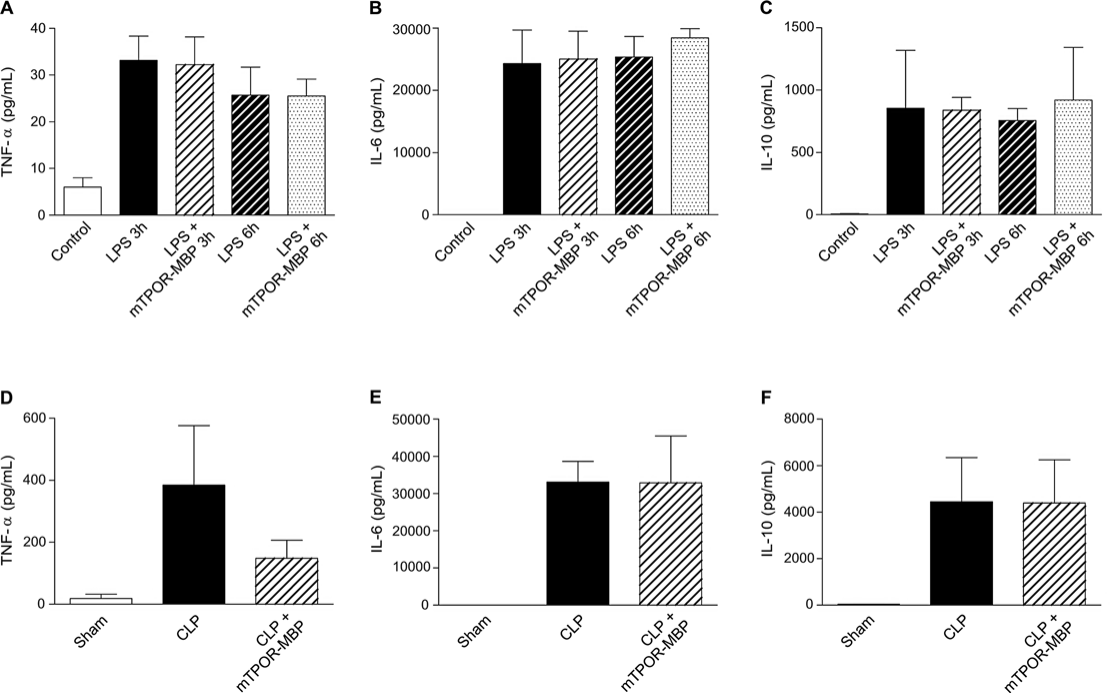
Plasma cytokine levels in the LPS model (upper panels) and in the cecal ligation and puncture (CLP) model (lower panels). Cytokine (TNF-α, IL-6, and IL-10) concentrations were measured in plasma samples (n = 5 per experimental group) at the indicated time-points in the LPS model and 18 hours after sepsis induction in the CLP model using a fluorescent bead–based multiplex immunoassay (Bio-plex®/Luminex®, Luminex Co., Austin, TX), as previously described [29, 30].

In severe sepsis pathophysiology, intense pro-inflammatory reactions, which includes the secretion and release of several cytokines in blood, are usually associated with the development of collateral tissue damage and consequent organ failure [31]. As expected, the induction of systemic inflammation or sepsis was associated with a relevant increase in circulating cytokine concentrations in both models studied (Fig 7). Plasma levels of TNF-α, IL-6, and IL-10 were strongly increased after 3 and 6 hours from LPS administration, as well as after sepsis induction in the CLP model, in comparison to those measured in saline-injected or sham-operated mice (Fig 7). Quite surprisingly though, mTPOR-MBP did not induce any significant change in cytokine levels neither in the LPS model at both time-points examined (Fig 7, panels A-C), nor in the CLP model, although a tendency toward a reduction in TNF-α levels was observed in this model (Fig 7, panels D-F).

mTPOR-MBP alone did not show any effect on cytokine levels (not shown).

### mTPOR-MBP modulates platelet-leukocyte adhesion in experimental endotoxemia and polymicrobial sepsis

Sepsis development has been associated with the appearance in the circulation of increased platelet-monocyte and platelet-neutrophil aggregates, which are believed to have a relevant role in the progression to the most severe forms of the disease [31]. In particular, platelet-monocyte adhesion is considered an early and sensitive measure of platelet activation [32], while platelet-neutrophil interaction has a crucial role in the recruitment of neutrophils to inflamed tissues and in the development of target organ injury [33]. Therefore, we studied the effects of mTPOR-MBP administration on platelet-leukocyte adhesion in both experimental models.

**Fig 8:**
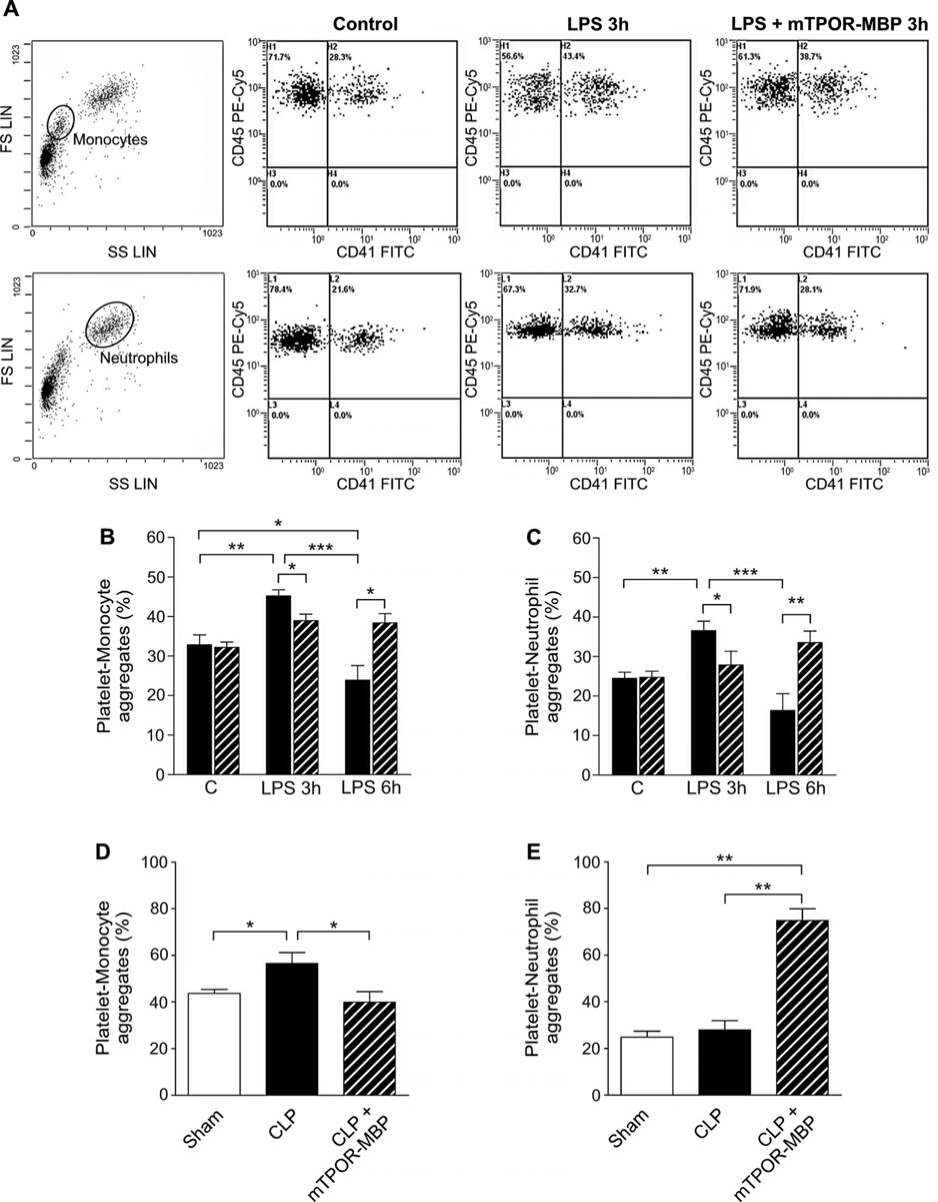
Effect of mTPOR-MBP on platelet-leukocyte adhesion in LPS model and in the cecal ligation and puncture (CLP) model. (A) Representative flow cytometry analysis of platelet-monocyte (upper lane) and platelet-neutrophil aggregates (lower lane) detected in mice injected with saline (Control) and in mice injected with LPS treated or not with mTPOR-MBP. The two left panels show an example of monocyte (upper panel) and neutrophil (lower panel) subpopulations, identified by size and granularity in the forward scatter (FS LIN) versus side scatter (SS LIN) dot plot, using a logic gate, over the total CD45-positive leukocyte population. The others panels are representative of CD45 versus CD41 dot plots obtained in the different experimental conditions. Platelet-monocyte and platelet-neutrophil aggregates were identified, within the logic gates previously selected, as CD45 and CD41 double-positive cells (see the Materials and Methods section for experimental details). (B-C) Quantification of the percentage of platelet-monocyte (panel B) and platelet-neutrophil (panel C) aggregates detected ex vivo by flow cytometry at the indicated time-points after LPS administration, in mice treated (dashed columns) or not (black columns) with mTPOR-MBP. The number of animals studied per experimental group was as follows: Control (C) = 10; mTPOR-MBP alone = 9; LPS 3h = 8; LPS 3h + mTPOR-MBP = 6; LPS 6h = 5; LPS 6h + mTPOR-MBP = 5. (D-E) Quantification of the percentage of platelet-monocyte (panel D) and platelet-neutrophil (panel E) aggregates detected ex vivo by flow cytometry in sham-operated mice (white columns) and septic mice 18 hours after CLP, treated (dashed columns) or not (black columns) with mTPOR-MBP. The number of animals studied per experimental group was as follows: Sham-operated mice = 5; CLP = 5; CLP + mTPOR-MBP = 5. * *P* < 0.05; ** *P* < 0.01; *** *P* < 0.001.

In the LPS model, both platelet-monocyte and platelet neutrophil aggregates increased at 3 hours, and decreased to levels lower than basal values at 6 hours from LPS injection (Fig 8, panels A-C). The temporal trend of platelet-leukocyte adhesion closely paralleled that of TPO release in the circulation of LPS-treated mice. Furthermore, we found a significant correlation between TPO levels and platelet-monocyte adhesion (r = 0.5185, *P*<0.05), and between TPO levels and platelet-neutrophil adhesion (r = 0.6805, *P*<0.001) measured ex vivo. mTPOR-MBP administration significantly blunted the increase in both platelet-monocyte and platelet-neutrophil adhesion observed at 3 hours from LPS administration (Fig 8, panels B and C). On the contrary, the percentage of both platelet-monocyte and platelet-neutrophil aggregates were higher in endotoxemic mice treated than in those untreated with mTPOR-MBP after 6 hours from LPS injection (Fig 8, panels B and C).

Analogously to what we observed in the LPS model, also in the CLP model platelet-monocyte adhesion was enhanced after sepsis induction in comparison to that measured in sham-operated mice (Fig 8D), showing a temporal trend similar to that of TPO release in the circulation. However, no correlation was found between TPO and platelet-monocyte adhesion in this model. Moreover, mTPOR-MBP administration was associated with a significant reduction in the percentage of platelet-monocyte aggregates, to levels similar to those detected in sham-operated mice (Fig 8D). On the contrary, platelet neutrophil binding in septic mice did not differ from the levels observed in sham-operated mice, at least at the time-point examined (Fig 8E), while the administration of mTPOR-MBP was associated with an evident increase in circulating platelet-neutrophil aggregates in septic mice (Fig 8E).

No change in platelet-leukocyte adhesion was detected in mice injected with saline or mTPOR-MBP alone in both experimental models.

**Fig 9:**
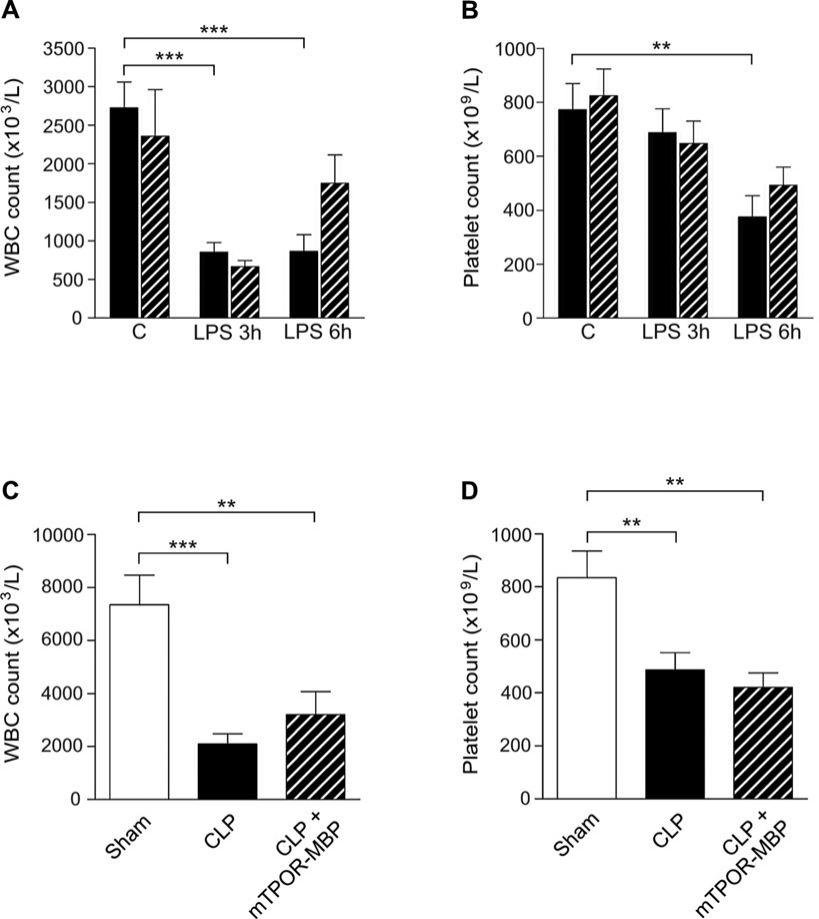
White blood cell (WBC) and platelet counts in LPS model and in the cecal ligation and puncture (CLP) model. Panel A and B show WBC and platelet counts, respectively, measured at the indicated time-points after LPS administration in mice treated (dashed columns) or not (black columns) with mTPOR-MBP. The number of animals studied per experimental group was as follows: Control (C) = 11; mTPOR-MBP alone = 8; LPS 3h = 11; LPS 3h + mTPOR-MBP = 10; LPS 6h = 6; LPS 6h + mTPOR-MBP = 8. Panel C and D show WBC and platelet counts, respectively, measured in sham-operated mice (white columns) and septic mice 18 hours after CLP, treated (dashed columns) or not (black columns) with mTPOR-MBP. The number of animals studied per experimental group was as follows: Sham-operated mice = 5; CLP = 11 CLP + mTPOR-MBP = 7. ** *P* < 0.01; *** *P* < 0.001.

Finally, the induction of systemic inflammation or sepsis was associated with a relevant decrease in leukocyte and platelet counts in both models, an effect that was neither observed in mice injected with saline or mTPOR-MBP alone, nor in sham-operated mice (Fig 9). The administration of mTPOR-MBP did not prevent the drop in leukocyte and platelet count at 3 hours from LPS injection, and had only a modest effect on leukocyte count, which resulted higher in LPS-treated mice than controls, but not on platelet count, at 6 hours (Fig 9, panels A and B). No effect on leukocyte and platelet count drop was observed after mTPOR-MBP administration in the CLP model either (Fig 9, panels C and D).

## Discussion

The aim of this study was to define the contribution of TPO in enhancing platelet-leukocyte adhesion associated with systemic inflammation and sepsis, and to explore the potential therapeutic effect of TPO blockade in inhibiting the development of organ damage in an acute endotoxemia model, which mimics a systemic inflammatory response syndrome, and in the CLP model, which is an abdominal infection model that leads to polymicrobial sepsis. Therefore, we synthesized a chimeric protein, mTPOR-MBP, and characterized it for its ability to inhibit in vivo the biological activity of TPO. We then tested its effects in these two widely used murine experimental models.

Until now, the involvement of TPO in the pathogenesis of sepsis and other acute critical diseases has remained controversial. Whereas several observations have suggested, in recent years, that TPO may be involved in the pathogenesis of sepsis and of other critical diseases, for instance acute lung injury and acute pancreatitis [3, 12, 15–18], some studies have rather sustained a beneficial effect of TPO administration in preventing organ damage [19, 20]. A first major finding of our study resides in the observation that mTPOR-MBP, by inhibiting TPO in vivo, reduced histological damage in target organs (lung, liver, and gut) in both models studied. This result confirms and further supports the hypothesis that TPO may represent a pathogenic mediator of organ damage in sepsis and other critical diseases, for instance burn injury [3].

In both experimental models, TPO and cytokine concentrations significantly increased after the induction of systemic inflammation or sepsis, confirming their resemblance, for this aspect, to what observed in patients [15, 16]. Furthermore, we observed an increase in platelet-monocyte aggregation in both models, with a temporal trend similar to that of TPO release in the bloodstream, which could suggest that TPO may be responsible for stimulating platelet activation and platelet-monocyte aggregation associated with the development of systemic inflammation and sepsis. This conclusion is further supported by the positive correlation we found between TPO and platelet-leukocyte adhesion in the LPS model. On the contrary, TPO and platelet-leukocyte adhesion did not correlate in the CLP model, at least at the time-point we examined. This discrepancy between the two models may be related to the differences in the model pathophysiology. The CLP model, indeed, closer reproduces the complex pathophysiology of human sepsis, associated with the activation of a number of host mediator systems [34], whereas endotoxemia induced by LPS injection, rather mimics a systemic inflammatory response syndrome, usefully allowing to more precisely study the time-course of the inflammatory events elicited by LPS. Therefore, it may be hypothesized that TPO may be involved in triggering the early phase of platelet-leukocyte adhesion, which could be later sustained, at least in sepsis, by other mediators.

The administration of mTPOR-MBP inhibited the increase of platelet-monocyte binding both in the LPS model at three hours and in the CLP model, definitively showing that high TPO levels represent a pivotal stimulus of platelet activation and platelet-monocyte aggregation in these two models. On the contrary, mTPOR-MBP administration was associated with the persistence of platelet-neutrophil aggregates in the circulation at levels higher than in untreated mice, both in the LPS model at 6 hours and in the CLP model. Several studies have previously shown that platelet activation and thrombocytopenia are associated with the development of organ dysfunction during sepsis and with increased sepsis severity [35–37]. Frequent abnormalities in platelet count have been, indeed, reported, and sepsis severity correlates with the decrease in platelet count [35–40]. Furthermore, circulating platelets are activated in experimental sepsis models, as well as in septic patients [35–37, 41–44], a phenomenon that can predispose to micro-thrombotic events, which may reduce organ perfusion and participate in microcirculatory dysfunction [45–50]. In addition, sepsis development has been associated with increased surface expression of platelet adhesion molecules, and increased circulating platelet-monocyte aggregates, which are considered not only a sensitive measure of platelet activation, but also have major pro-inflammatory and pro-thrombotic consequences [32]. Finally, a link between platelet-leukocyte, in particular platelet-neutrophil adhesion, and clinical outcome has been reported in septic patients [33, 42–44]. Platelet-neutrophil aggregates, which are required for the recruitment of neutrophils to inflamed tissues [33], increase, indeed, upon sepsis development, but decrease in those patients who develop organ failure and eventually die [42, 44]. This phenomenon is considered expression of the increased recruitment of neutrophils to inflamed tissues and peripheral sequestration, which may be implicated in the development of sepsis-induced multiple organ dysfunction syndrome [33, 42, 44]. Therefore, the increase of platelet-neutrophil adhesion observed in our study at later time-points after mTPOR-MBP administration may reflect the inhibition of platelet activation and adhesion to leukocytes and, consequently, of platelet-neutrophil aggregates to the endothelium, which may be implicated in the protective effect of TPO blockade on inflammation- or sepsis-induced organ-damage. These results are in agreement with those of previous experimental studies that suggest that inhibiting platelet activation may reduce sepsis-related mortality. For instance, platelet depletion had a protective effect in CLP model [51–53], and the modulation of platelet activation by using specific anti-P-selectin antibody [53] or drugs that inhibit platelet activation, such as clopidogrel, was beneficial [54] both in endotoxin shock [54] and polymicrobial sepsis [55], leading to hypothesize that anti-platelet drugs may represent a potential treatment in sepsis or acute respiratory distress syndrome [56].

In our study, we observed a dramatic reduction in leukocyte and platelet counts in both models, which was not prevented by mTPOR-MBP. Therefore, this well-described phenomenon is not related to TPO activity, but rather to the direct action of LPS on leukocytes, and, may be, platelets themselves. However, this observation excludes that the decrease in platelet-leukocyte aggregation we detected with mTPOR-MBP may be caused by the drop in leukocyte and platelet absolute counts.

Quite surprisingly, mTPOR-MBP was not able to inhibit the increase in plasma concentrations of TNF-α, IL-6 and IL-10 observed in both models, a well-known phenomenon that participates in the initiation and progression of organ damage during systemic inflammation and sepsis [31]. Therefore, our data shows that TPO inhibition by mTPOR-MBP exerts its protective effect on organ damage primarily by influencing platelet-leukocyte adhesion, while it does not affect cytokine release. Similar results, i.e. a dissociation between the protective effect on sepsis-related mortality and cytokine increased production, were previously described, in a model of endotoxin shock, with the administration of erythropoietin, another hematopoietic growth factor/cytokine [57]. This observation has interesting implications for our knowledge of sepsis pathogenesis, since it suggests that the development of sepsis-induced organ damage and cytokine release may not be necessarily related. However, our experimental data cannot exclude that the inhibition by mTPOR-MBP of other TPO actions, for instance the ability to stimulate the production of oxygen radicals or IL-8 from leukocytes, or to modulate apoptosis in several cell types [3], may also have had a role in the effects observed in our study. Finally, another intriguing hypothesis may be evoked to explain the protective effect of TPO blockade in these experimental models. We have previously shown that high TPO concentrations in plasma samples from patients with septic shock cooperate with TNF-α and IL-1β in depressing myocardial contractility [12]. Therefore, we could hypothesize that blocking TPO in vivo by a selective antagonist as mTPOR-MBP could inhibit the priming effect of TPO on the activity of other mediators, such as inflammatory cytokines, thus dampening the development of damage to target organs.

We cannot precisely define the origin of the rise in TPO levels observed in our study after the induction of acute endotoxemia and polymicrobial sepsis. Platelet mass is considered the primary regulator of TPO levels [4], and both endotoxemic and septic animals had a profound thrombocytopenia, together with increased indices of ex vivo platelet activation. Platelets themselves, which are known to release active TPO upon stimulation [58], could also be a major source for TPO release. Finally, high TPO levels may derive from increased hepatic synthesis, since IL-6 enhances TPO synthesis from hepatocytes [59]. However, this last possibility can be excluded in the LPS model, on the basis of the kinetic of TPO appearance in mouse plasma, too rapid to implicate protein synthesis.

Several limits have to be taken into account in interpreting our results. First, we did nor evaluate the effect of TPO inhibition on long-term organ protection, neither on survival and recovery in both models. Therefore, we cannot exclude that the observed effects of mTPOR-MBP administration are transient, and/or that TPO inhibition has not a significant impact on disease evolution. Second, we cannot provide a clear demonstration of the mechanistic effect of TPO in the pathophysiology of systemic inflammation or sepsis. Although intriguing, the link between the observed effects of TPO inhibition on platelet-leukocyte interaction and the development of inflammation- or sepsis-associated tissue damage in target organs still remains not definitely proven by our experiments.

In conclusion, blockade of TPO biological activity significantly reduced the severity of organ damage in both experimental endotoxemia and polymicrobial sepsis. This protective effect is associated with the inhibition of the increase in platelet-leukocyte adhesion, but not of cytokine release, observed during systemic inflammation and sepsis. Our results support the need to further explore the possibility of inhibiting TPO in the intent to prevent or reduce organ damage in patients affected by systemic inflammatory response or sepsis.
